# Mg^2+^ and Cr^3+^ Co-Doped LiNi_0.5_Mn_1.5_O_4_ Derived from Ni/Mn Bimetal Oxide as High-Performance Cathode for Lithium-Ion Batteries

**DOI:** 10.3390/nano15060429

**Published:** 2025-03-11

**Authors:** Dehua Ma, Jiawei Wang, Haifeng Wang, Guibao Qian, Xingjie Zhou, Zhengqing Pei, Kexin Zheng, Qian Wang, Ju Lu

**Affiliations:** 1 College of Materials and Metallurgy, Guizhou University, Guiyang 550025, China; mdehua919@gmail.com (D.M.); jwwang@gzu.edu.cn (J.W.); 2414902764@163.com (G.Q.); 1916331338@163.com (X.Z.); 3163429870@163.com (Z.P.); 122754931@163.com (K.Z.); 1169979509@163.com (Q.W.); 2642656692@163.com (J.L.); 2 Guizhou Key Laboratory of Metallurgical Engineering and Process Energy Conservation, Guiyang 550025, China; 3 Engineering Technology and Research Center of Manganese Material for Battery, Tongren 554300, China

**Keywords:** cathode material, LiNi_0.5_Mn_1.5_O_4_, Ni/Mn bimetal oxide, co-doping

## Abstract

In this study, pure and Mg^2+^/Cr^3+^ co-doped Ni/Mn bimetallic oxides were used as precursors to synthesize pristine and doped LNMO samples. The LNMO samples exhibited the same crystal structure as the precursors. XRD analysis confirmed the successful synthesis of LNMO cathode materials using Ni/Mn bimetallic oxides as precursors. FTIR and Raman spectroscopy reveal that Mg^2+^/Cr^3+^ co-doping promotes the formation of the Fd3m disordered phase, effectively reducing electrochemical polarization and charge transfer resistance. Furthermore, co-doping significantly lowers the Mn^3+^ content on the LNMO surface, thereby mitigating Mn^3+^ dissolution. Significantly, Mg^2+^/Cr^3+^ co-doping induces the emergence of high-surface-energy {100} crystal facets in LNMO grains, which promote lithium-ion transport and, finally, enhance rate capability and cycling performance. Electrochemical analysis indicates that the initial discharge capacities of LNMO-0, LNMO-0.005, LNMO-0.010, and LNMO-0.015 were 126.4, 125.3, 145.3, and 138.2 mAh·g^−1^, respectively, with capacity retention rates of 82.45%, 82.93%, 83.32%, and 82.08% after 100 cycles. Furthermore, the impedance of LNMO-0.010 prior to cycling was 97.38 Ω, representing a 14.35% reduction compared to the pristine sample. After 100 cycles, its impedance was only 58.61% of that of the pristine sample, highlighting its superior rate capability and cycling stability. As far as we know, studies on the synthesis of LNMO cathode materials via the design of Ni/Mn bimetallic oxides remain limited. Accordingly, this work provides an innovative approach for the preparation and modification of LNMO cathode materials. The investigation of Ni/Mn bimetallic oxides as precursors, combined with co-doping by Mg^2+^ and Cr^3+^, for the synthesis of high-performance LiNi_0.5_Mn_1.5_O_4_ (LNMO) aims to provide insights into improving rate capability, cycling stability, reducing impedance, and enhancing capacity retention.

## 1. Introduction

In recent years, lithium-ion batteries (LIBs) have found widespread application in portable smart devices, electric vehicles, plug-in hybrid vehicles, and energy storage systems due to their superior energy density and cycle life [1,2,3,4,5,6]. To meet the growing demands for enhanced energy density, extended cycle life, and high-rate performance in these applications, it is crucial to improve the performance of LIBs, which is primarily determined by the choice of cathode materials [7]. Among various lithium battery cathodes, the high-voltage spinel LNMO has emerged as one of the most promising candidates for next-generation high energy density LIBs. This is attributed to its high operating voltage (~4.7 V), substantial energy density (658 Wh∙kg^−1^), and three-dimensional lithium-ion diffusion pathways [8,9,10].

Despite these advantages, the electrochemical performance of synthesized LNMO encounters several significant challenges. The manganese (Mn) within LNMO can dissolve in the electrolyte at elevated temperatures through the disproportionation reaction 2Mn^3+^→Mn^2+^ + Mn^4+^, leading to Jahn–Teller distortion that alters the crystal structure. This alteration compromises the structural integrity of the cathode material, thereby diminishing its stability [11,12,13,14,15]. Moreover, at the high operating voltage of 4.7 V for LNMO, side reactions such as electrolyte decomposition are likely to occur at the electrode/electrolyte interface [16]. These side reactions lead to the formation of a cathode/electrolyte interphase (CEI) layer, which in turn increases the interfacial membrane resistance [17]. Strategies such as ion doping and surface coating have been demonstrated to effectively mitigate these challenges [18,19,20]. However, studies on Mg^2+^/Cr^3+^ co-doping in LNMO are relatively scarce, and the synergistic effects of Mg^2+^ and Cr^3+^ remain unclear.

The arrangement of Ni and Mn atoms within the spinel structure differs, resulting in LNMO materials that consist of both the disordered Fd3m phase and the ordered P4_3_32 phase with distinct spatial symmetry [21]. In the P4_3_32 ordered structure, Mn ions exhibit a valence state of +4, while in the Fd3m disordered structure, both Mn^3+^ and Mn^4+^ coexist. The presence of Mn^3+^ facilitates ionic transport, leading to ionic conductivity in the Fd3m disordered phase that is two orders of magnitude higher than that of the P4_3_32 ordered phase, thereby enhancing the rate performance of LNMO [22]. However, excessive Mn^3+^ presence can negatively impact the cycle life of the battery [23]. Moreover, during the battery’s charge/discharge process, compared to the two-phase transition in the P4_3_32 ordered structure, the Fd3m disordered structure undergoes a single-phase transition, which experiences less mechanical stress and benefits the cycling stability of the material [24,25]. Consequently, synthesizing LNMO materials with a higher proportion of the Fd3m disordered phase and appropriate Mn^3+^ content can simultaneously enhance both rate capability and cycling performance.

The general formula for bimetal oxides is AB_2_O_4_, where A represents divalent cations such as Mg^2+^, Fe^2+^, or Ni^2+^, and B represents trivalent cations such as Mn^3+^, Cr^3+^, or Al^3+^. These oxides share the Fd3m space’s group spinel structure with LNMO [26,27]. Bimetal oxides exhibit excellent performance in various fields [28]. Utilizing Ni/Mn bimetal oxides as a doping template facilitates ion doping and mitigates the stress effects associated with crystal structure changes during the calcination process for LNMO synthesis. Furthermore, in NiMn_2_O_4_ (NMO), both tetrahedral and octahedral sites can be occupied by Ni and Mn cations, leading to cation disorder within the oxide [29,30]. Importantly, NiMn_2_O_4_ exhibits a porous composite structure that enhances the intercalation and deintercalation of lithium-ions, promoting rapid ion transport. Consequently, utilizing Ni/Mn bimetal oxides as precursors for the synthesis of both LNMO and doped LNMO constitutes an effective and feasible strategy. As far as we know, the preparation of LNMO cathodes using Ni/Mn bimetal oxide precursors has rarely been investigated.

In this study, NH_3_·H_2_O was utilized as a complexing agent and precipitant to achieve a uniform mixing of Ni and Mn metal ions in solution. Air was subsequently introduced into the system to oxidize the metal hydroxides into metal oxide precipitates, which were then pre-calcined to obtain Ni/Mn bimetal oxides. The resultant Ni/Mn bimetal oxides were mixed with Li_2_CO_3_ in stoichiometric ratios and calcined, leading to the transformation of the Ni/Mn bimetal oxides into LNMO. To further enhance the electrochemical performance of LNMO, Mg^2+^/Cr^3+^ co-doped Ni/Mn bimetal oxide precursors were synthesized, resulting in a series of co-doped LiNi_0.5-x/2_Mg_x/2_Cr_x/2_Mn_1.5-2/x_O_4_ (x = 0, 0.005, 0.01, 0.015). The results confirm the successful synthesis of the LNMO cathode material, with ionic co-doping effectively facilitating the formation of the Fd3m disordered structure and suppressing Mn^3+^ content. The co-doped LNMO demonstrates significantly improved cycling stability and rate performance.

## 2. Experimental Section

### 2.1. Material Synthesis

**Scheme 1:**
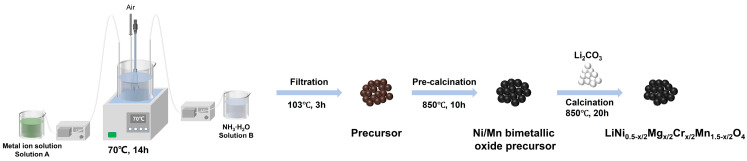
Synthesis process of LiNi_0.5-x/2_Mg_x/2_Cr_x/2_Mn_1.5-x/2_O_4_ (x = 0, 0.005, 0.01, 0.015) samples.

The specific preparation process is detailed in Scheme 1, where the pristine and co-doped LiNi_0.5-x/2_Mg_x/2_Cr_x/2_Mn_1.5-x/2_O_4_ (x = 0, 0.005, 0.010, 0.015) was synthesized through liquid-phase oxidation followed by two-stage calcination. Initially, (0.5-x/2) mol of NiSO_4_·6H_2_O, (1.5-x/2) mol of MnSO_4_·H_2_O, (x/2) mol of MgSO_4_, and (x/2) mol of CrH_3_O_12_S_3_ were mechanically stirred and dissolved in 1000 mL of deionized water, designated solution A. Subsequently, NH_3_·H_2_O was mixed with deionized water in a 1:1 ratio to serve as a complexing and precipitating agent, referred to as solution B. A total of 500 mL of deionized water was added to the reactor, which was heated in a water bath while continuously supplying air until the temperature reached 70 °C. Solutions A and B were introduced into the reactor at rates of 1.33 mL∙min^−1^ and 2.71 mL∙min^−1^, respectively, and the reaction was maintained for 14 h. Upon completion of the reaction, the black-brown precipitate was filtered, washed several times with deionized water, and dried at 100 °C for 3 h. The precursor was subjected to calcination at 500 °C in a muffle furnace for 10 h to synthesize Ni/Mn bimetallic oxide precursors, designated NMO-0, NMO-0.005, NMO-0.01, and NMO-0.015, corresponding to varying doping content. This precursor was uniformly mixed with stoichiometric Li_2_CO_3_ (with an excess of 5 wt% to compensate for lithium loss) and calcined at 850 °C for 20 h. After natural cooling to room temperature, the powder was collected to obtain the product LiNi_0.5-x/2_Mg_x/2_Cr_x/2_Mn_1.5-x/2_O_4_ (x = 0, 0.005, 0.01, 0.015). The resulting products were designated according to the co-doping content: LNMO-0, LNMO-0.005, LNMO-0.01, and LNMO-0.015.

### 2.2. Material Characterization and Electrochemical Analysis

All detailed information for the material characterization, computational methods, and electrochemical measurements are shown in the Supporting Information.

## 3. Results and Discussion

**Figure 1:**
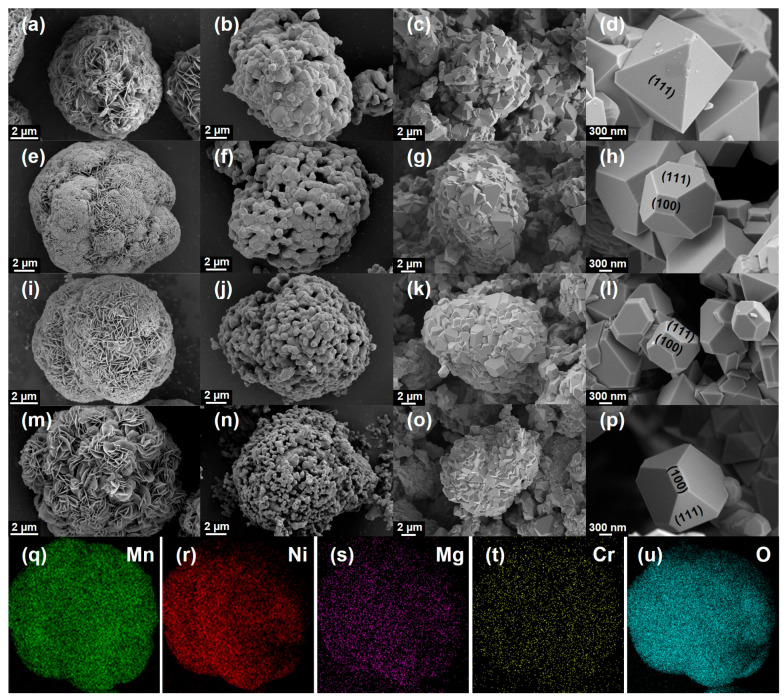
Morphological evolution during the preparation of pristine and Mg^2+^/Cr^3+^ co-doped LNMO. (**a**–**c**) Morphologies of the pristine precursor, NMO-0, and LNMO-0; (**d**) particle morphology of LNMO-0; (**e**–**g**) morphologies of the precursor, NMO-0.005, and LNMO-0.005 in 0.005 Mg^2+^/Cr^3+^ co-doped samples; (**h**) particle morphology of LNMO-0.005; (**i**–**k**) morphologies of the precursor, NMO-0.010, and LNMO-0.010 in 0.010 Mg^2+^/Cr^3+^ co-doped samples; (**l**) particle morphology of LNMO-0.010; (**m**–**o**) morphologies of the precursor, NMO-0.015, and LNMO-0.015 in 0.015 Mg^2+^/Cr^3+^ co-doped samples; (**p**) particle morphology of LNMO-0.015; (**q**–**u**) EDS mapping of 0.010 Mg^2+^/Cr^3+^ co-doped precursor.

The morphology of the pristine and Mg^2+^/Cr^3+^ co-doped LNMO during the preparation process was characterized using scanning electron microscopy (SEM). As illustrated in Figure 1a,e,i,m, the morphology of the pristine and lightly co-doped precursors consists of nanosheets that adhere to and grow on the surfaces of nanospheres, whereas the precursor with 0.015 Mg^2+^/Cr^3+^ co-doping is entirely composed of nanosheets. With an increasing Mg^2+^/Cr^3+^ content, the size of the nanospheres diminishes, suggesting that Ni and Mn in the precursor exist as more micro-aggregated nanosheets. LNMO precursors prepared via coprecipitation generally exhibit a more disordered spherical morphology [31]. In contrast, the morphology obtained through this method, characterized by the coexistence of nanosheets and nanospheres, is more organized and provides an increased specific surface area for enhanced reactivity. Energy-dispersive spectroscopy (EDS) was employed to assess the chemical composition and elemental distribution of both the pristine and co-doped precursors. Figure S1a–m and Figure 1q–u reveal the presence of Ni, Mn, and O in the pristine precursor, while the co-doped Mg^2+^/Cr^3+^ precursor includes Ni, Mn, Mg, Cr, and O. EDS mapping demonstrates that Mg and Cr are uniformly distributed throughout the doped precursor, confirming the successful incorporation of Mg^2+^ and Cr^3+^ ions into the precursor.

**Figure 2:**
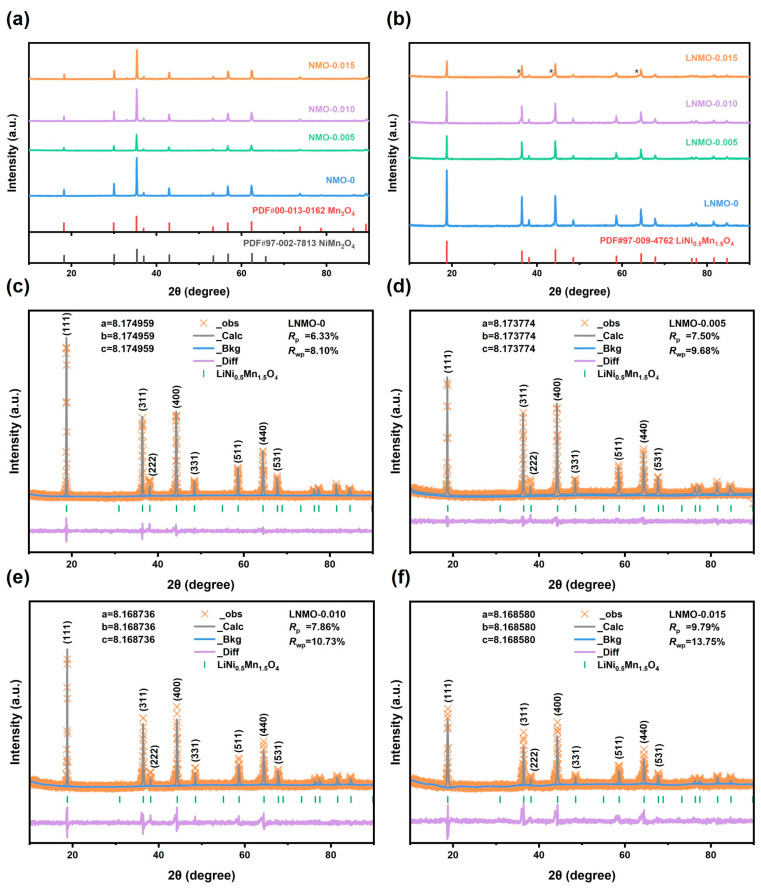
Structural analysis of pristine and Mg^2+^/Cr^3+^ co-doped NMO and LNMO, including the Rietveld refinement results for both pristine and Mg^2+^/Cr^3+^ co-doped LNMO. (**a**) XRD patterns of pristine and Mg^2+^/Cr^3+^ co-doped NMO; (**b**) XRD patterns of pristine and Mg^2+^/Cr^3+^ co-doped LNMO; (**c**–**f**) Rietveld refinement results for pristine and Mg^2+^/Cr^3+^ co-doped LNMO.

Figure 1b,f,j,n illustrates the morphologies of the pristine and co-doped NMO precursors. All four samples consist of numerous small octahedral structures that aggregate into spherical shapes. As the Mg^2+^/Cr^3+^ content increases, the porosity of the spherical octahedral aggregates also increases, leading to a higher specific surface area. Figure S2 and Table S1 indicate that increasing the content of Mg^2+^/Cr^3+^ would enhance the BET surface area, pore volume, and average pore diameter of the NMO precursor, which is consistent with the SEM results. However, the data for NMO-0.010 show a downward trend, which is due to the fact that the voids in the morphology of NMO-0.005 are concentrated and relatively large, while the octahedral structure size of NMO-0.015 becomes smaller. The morphology of NMO-0.010 exhibits a uniform octahedral structure size and an even distribution of voids within the spherical aggregates, which can provide more active sites and diffusion pathways, potentially enhancing the capacity of LNMO. The structures of both pristine and co-doped NMO, as well as LNMO, were analyzed using X-ray diffraction (XRD), and the results for LNMO were subjected to Rietveld refinement. As shown in Figure 2a, the main diffraction peaks of all samples align perfectly with the spinel-type structures of NiMn_2_O_4_ (PDF#97-002-7813) and Mn_3_O_4_ (PDF#00-013-0162) in the Fd3m space group. It is observed that as the Mg^2+^/Cr^3+^ co-doping content increases, the XRD signals gradually shift toward higher angles, indicating the successful incorporation of Mg^2+^ and Cr^3+^ into the NMO precursor. Given that the ionic radius of Cr^3+^ (0.61 Å) is smaller than that of Mn^3+^ (0.65 Å), the crystal volume decreases [32]. The emergence of the Mn_3_O_4_ phase results from the stoichiometric ratio of Ni to Mn in solution A being 1:3, while in the bimetallic oxide NiMn_2_O_4_, the ratio is 1:2. This discrepancy leads to the calcination of excess Mn that cannot bond with Ni, resulting in the formation of Mn_3_O_4_.

Figure 1c,g,k,o illustrates the morphologies of the pristine and co-doped LNMO samples. All four samples consist of numerous small octahedral crystals that aggregate into spherical shapes, resembling the spherical octahedral aggregates of the NMO precursor; however, they exhibit fewer voids and a more compact arrangement of the octahedral crystals. The spherical aggregation in LNMO-0 and LNMO-0.005 is less pronounced, with the octahedral crystals appearing fragmented and disordered, accompanied by a significant amount of surface impurities. In contrast, LNMO-0.010 and LNMO-0.015 display more pronounced spherical aggregation and cleaner surfaces, free from other impurities. The smaller sizes of the spherical aggregates and octahedral crystals in LNMO-0.015 are attributed to the micro-aggregated nanosheet precursors and the smaller octahedral crystal size observed in the NMO-0.015 precursor. LNMO-0.010 exhibits a favorable morphology with clear spherical aggregation and larger, uniformly distributed octahedral crystals. The larger particles may reduce the contact area between the cathode material and the electrolyte, enhancing structural stability [33,34]. Figure 1d,h,l,p presents the crystal morphologies of LNMO-0, LNMO-0.005, LNMO-0.010, and LNMO-0.015. Notably, the morphology of the primary particles exhibits intriguing variations with increasing Mg^2+^/Cr^3+^ co-doping content. The crystal surface orientations correspond to the established {111} plane orientation of the octahedral face-centered cubic framework and their truncated surface orientations [35,36]. The primary particles of LNMO-0 are composed of {111} crystal facets, while the grains of LNMO-0.005, LNMO-0.010, and LNMO-0.015 form new {100} crystal facets at the truncated corners of the octahedral structure. Previous studies indicate that when doping with Cr^3+^ and calcining at temperatures above 800 °C, the {100} crystal planes, which possess higher surface energy, tend to grow preferentially over the {111} planes [37,38,39]. This suggests that during the calcination of NMO-0, the low-energy {111} planes preferentially grow, resulting in the sacrifice of the {100} and other higher-energy planes [40,41,42]. In contrast, after co-doping with Mg^2+^/Cr^3+^, the lattice structure becomes more stable, allowing for greater exposure of high-energy crystal planes during calcination. Among these, the LNMO-0.010 sample exhibits the highest exposure of high-energy crystal planes, which may facilitate the lithium-ion insertion/extraction process, thereby ensuring improved specific capacity and rate performance.

Figure 2b presents the XRD patterns for the synthesis of LiNi_0.5-x/2_Mg_x/2_Cr_x/2_Mn_1.5-x/2_O_4_ (x = 0, 0.005, 0.01, 0.015). The main diffraction peaks of all samples align with the LiNi_0.5_Mn_1.5_O_4_ structure in the Fd3m space group (PDF#97-009-4762). Remarkably, the diffraction peaks for LNMO-0 and LNMO-0.010 exhibit enhanced intensity and sharpness, indicating a higher degree of crystallinity, which is advantageous for improving charge/discharge capacity and cycling performance [43,44]. As the co-doping content of Mg^2+^ and Cr^3+^ increases, a gradual shift of XRD signals toward higher angles is observed, confirming the successful incorporation of these ions into the NMO precursor, leading to a reduction in crystal volume. The LNMO-0.015 sample shows weaker diffraction peaks at 2θ = 35°, 43.5°, and 63.5°, which can be attributed to the presence of rock salt impurity phases (Ni_x_O, Ni_6_MnO_8_, or Li_1-x_Ni_x_O, marked with *) [45,46]. This phase indicates that, during high-temperature calcination, some Mn in LNMO-0.015 is reduced from Mn^4+^ to Mn^3+^ due to lithium volatilization, maintaining charge balance and leading to the formation of disordered phases with an increased Mn^3+^ content [47]. Figure 2c,d,e,f depicts the Rietveld refinement results for the pristine and Mg^2+^/Cr^3+^ co-doped LNMO samples based on the Fd3m space group, where the 8a site is occupied by Li, the 16d sites are occupied by Ni and Mn, and the 32e sites are occupied by O. The lower R_wp_ values obtained in all refinements indicate a high level of agreement between the measured and fitted values. Table 1 summarizes the refined structural parameters, showing a decreasing trend in crystal parameters, which is consistent with the SEM results. It can be inferred that Mg^2+^/Cr^3+^ co-doping contributes to the reduction of crystal structure parameters; specifically, the ionic radius of Cr^3+^ (0.61 Å) is smaller than that of Ni^2+^ (0.69 Å), leading to a decrease in crystal volume. Furthermore, this is mainly ascribed to fewer oxygen deficiencies caused by the higher bond strengths of Mg/O (389 kJ∙mol^−1^) and Cr/O (461 kJ∙mol^−1^) compared with Ni/O (382 kJ∙mol^−1^) and Mn/O (402 kJ∙mol^−1^), in view of the larger ionic radius of Mn^3+^ than that of Mn^4+^ [48]. Additionally, the stronger Mg/O and Cr/O bonds contribute to the decrease in crystal volume, thereby reducing the lattice parameters. The I_311_/I_400_ ratio serves as an indicator of the distortion degree within the cubic spinel structure and reflects the stability of the crystal structure [49,50]. A smaller I_311_/I_400_ ratio signifies a more stable structure and enhanced cycling performance for the LNMO materials. As indicated in Table 1, the I_311_/I_400_ ratio decreases with Mg^2+^/Cr^3+^ co-doping, suggesting that the crystal structures of the co-doped samples exhibit greater stability, which supports the selection of more high-energy crystal planes during the calcination process of the samples with Mg^2+^/Cr^3+^ co-doping.

**Figure 3:**
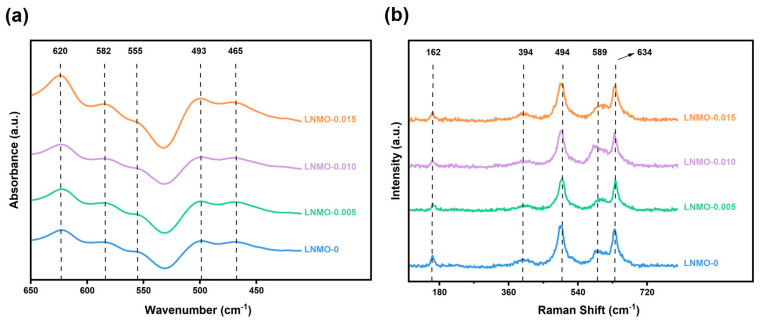
FTIR and Raman spectra of the pristine and Mg^2+^/Cr^3+^ co-doped LNMO. (**a**) FTIR; (**b**) Raman spectra.

LNMO materials are generally a mixture of the ordered P4_3_32 and disordered Fd3m structures, with the proportion of the Fd3m space group playing a key role in achieving superior electrochemical performance. Studies indicate that the Fd3m disordered structure offers enhanced electrochemical properties due to its greater charge transport capacity [24]. Since XRD analysis alone cannot reliably distinguish between the Fd3m and P4_3_32 space groups in synthesized LNMO samples [51], FTIR and Raman spectra were employed to assess the Ni/Mn ordering in both pristine and Mg^2+^/Cr^3+^ co-doped LNMO, as shown in Figure 3. According to Amatucci et al. [52], the P4_3_32 ordered structure presents eight infrared absorption bands in the range of 400–700 cm^−1^, whereas the Fd3m disordered structure, due to peak broadening, exhibits only five absorption bands. The FTIR for LNMO-0, LNMO-0.005, LNMO-0.010, and LNMO-0.015 in Figure 3a reveals five absorption bands at 620, 582, 555, 493, and 465 cm^−1^, indicative of the Fd3m disordered structure. Peaks observed at 620, 582, 555, and 493 cm^−1^ correspond to the Fd3m disordered structure, while the 465 cm^−1^ peak is associated with the P4_3_32 ordered structure [53]. Additionally, the signals at 620 cm^−1^ and 582 cm^−1^ correspond to symmetric stretching vibrations of Mn/O and Ni/O [10]. The FTIR intensity ratio at 620 cm^−1^ and 582 cm^−1^ (I_620_/I_582_) qualitatively reflects the disorder level in LNMO, with a higher I_620_/I_582_ ratio suggesting increased Ni/Mn disorder. From Figure 3a, the I_620_/I_582_ values for LNMO-0, LNMO-0.005, LNMO-0.010, and LNMO-0.015 are 1.43, 1.45, 1.56, and 2.80, respectively, indicating that Mg^2+^/Cr^3+^ co-doping partially substitutes Ni^2+^ and Mn^4+^, enhancing Ni/Mn disorder at the octahedral 16d sites and increasing the proportion of the Fd3m disordered structure. This trend aligns with the improved electrochemical performance observed upon Mg^2+^/Cr^3+^ co-doping. The Raman spectra of LNMO-0, LNMO-0.005, LNMO-0.010, and LNMO-0.015 are shown in Figure 3b. Mn/O stretching is observed at 634 cm^−1^ [54], while Ni^2+^/O stretching modes appear at 394 and 494 cm^−1^ [55]. Compared to the doped LNMO samples, LNMO-0 displays a pronounced peak at 162 cm^−1^, a feature of the P4_3_32 space group attributed to the reduced symmetry from Ni/Mn ordering [56] and the P4_3_32 polymorph [57,58]. This peak’s intensity diminishes significantly following Mg^2+^/Cr^3+^ co-doping, suggesting an increased disorder in LNMO. Additionally, the absence of distinct peak splitting in the 589–634 cm^−1^ range aligns with the Fd3m disordered structure. Thus, FTIR and Raman analyses confirm that all samples primarily exhibit the Fd3m disordered space group, with only a minor presence of the P4_3_32 ordered structure.

**Figure 4:**
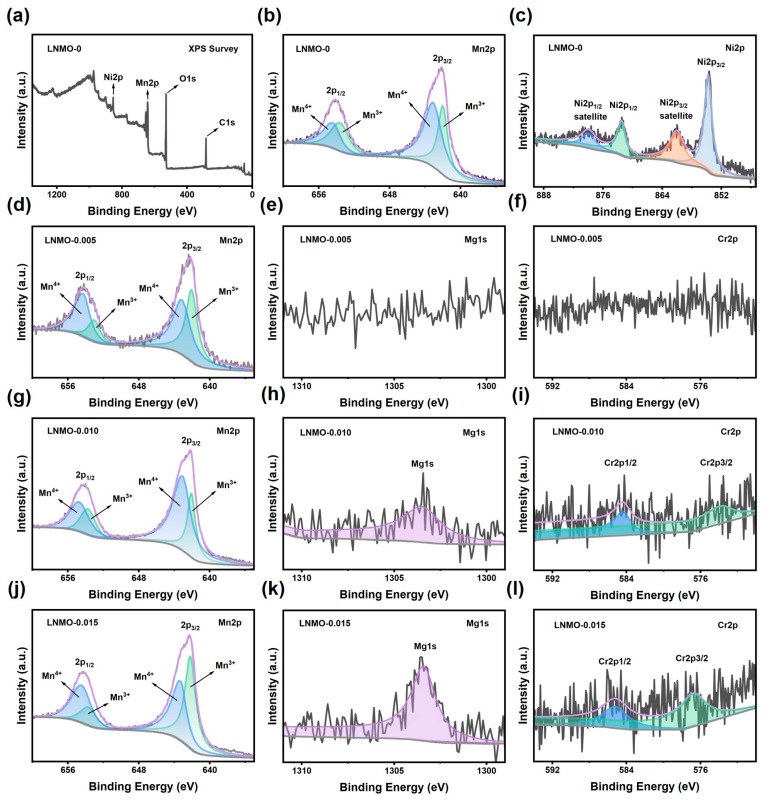
XPS spectra of pristine and Mg^2+^/Cr^3+^ co-doped LNMO. (**a**–**c**) Survey spectra, Mn 2p, and Ni 2p XPS spectra of LNMO-0; (**d**–**f**) Mn, Mg, and Cr XPS spectra of LNMO-0.005; (**g**–**i**) Mn, Mg, and Cr XPS spectra of LNMO-0.010; (**j**–**l**) Mn, Mg, and Cr XPS spectra of LNMO-0.015.

In order to investigate the impact of Mg^2+^/Cr^3+^ co-doping on the surface elemental distribution, X-ray Photoelectron Spectroscopy (XPS) was employed to analyze the valence states of elements in both bare and Mg^2+^/Cr^3+^ co-doped LNMO. Figure 4a shows the full XPS spectrum of LNMO-0, revealing the presence of Mn, Ni, O, and C on the surface. Figure 4b,d,g,j depicts the Mn2p spectra for LNMO-0, LNMO-0.005, LNMO-0.010, and LNMO-0.015, respectively, all of which can be deconvoluted into two peaks. The characteristic peaks at approximately ~642.0 eV and ~653.7 eV correspond to Mn^3+^, while those at around ~643.2 eV and ~654.5 eV indicate Mn^4+^, confirming the simultaneous presence of both Mn^3+^ and Mn^4+^ ions on the surfaces of all four samples [59]. Figure 4c and Figure S3a–c display the Ni2p spectra for LNMO-0, LNMO-0.005, LNMO-0.010, and LNMO-0.015, where four characteristic peaks can be observed. The peaks at approximately ~854.7 eV and ~872.7 eV correspond to the main peaks of Ni^2+^ in the Ni 2p_3/2_ and Ni 2p_1/2_ states, respectively. The observed characteristic peaks at approximately ~861.2 eV and ~879.5 eV correspond to the satellite peaks of Ni2p_3/2_ and Ni2p_1/2_, respectively, attributed to the multiple splitting of energy levels in nickel oxide (NiO) [59]. These features confirm that Ni in all four samples exists in the +2 oxidation state. Figure 4e,h,k presents the Mg1s spectra for LNMO-0.005, LNMO-0.010, and LNMO-0.015. Remarkably, no Mg1s characteristic peak is observed in LNMO-0.005, likely due to the low doping content of Mg^2+^ being below the detection limit of XPS. However, the presence of Mg^2+^ is confirmed in LNMO-0.010 and LNMO-0.015, with Mg1s characteristic peaks appearing at approximately ~1303.5 eV [10,60]. Figure 4f,i,l illustrates the Cr2p spectra for LNMO-0.005, LNMO-0.010, and LNMO-0.015. The absence of Cr2p peaks in LNMO-0.005 is attributed to the Cr^3+^ doping content being below the detection threshold. In LNMO-0.010 and LNMO-0.015, the characteristic peaks for Cr2p_3/2_ and Cr2p_1/2_ appear at approximately ~575.1 eV and ~585.2 eV, respectively, confirming the presence of Cr^3+^ [2,61]. The characteristic peaks of Mg1s and Cr2p validate the successful doping of Mg^2+^/Cr^3+^. Furthermore, the peak areas of Mg1s and Cr2p in LNMO-0.015 are greater than those in LNMO-0.010, indirectly indicating a higher co-doping content of Mg^2+^/Cr^3+^ in LNMO-0.015. Since the dissolution of Mn^2+^ and Mn^4+^ is generally associated with the disproportionation of Mn^3+^, a higher Mn^3+^ content can negatively impact the cycling performance of LNMO materials. Therefore, reducing the Mn^3+^ content in the surface region may alleviate the dissolution of Mn^2+^ and Mn^4+^, thus enhancing the cycling performance of LNMO. The relative content of Mn^3+^ can be quantified based on the peak area ratio of Mn2p_3/2_ [62]. As observed in Figure 4b,d,g,j, the Mn^3+^ content on the particle surface decreases with 0.010 Mg^2+^/Cr^3+^ co-doping, whereas it exceeds that of the undoped sample at a doping content of 0.015.

**Figure 5:**
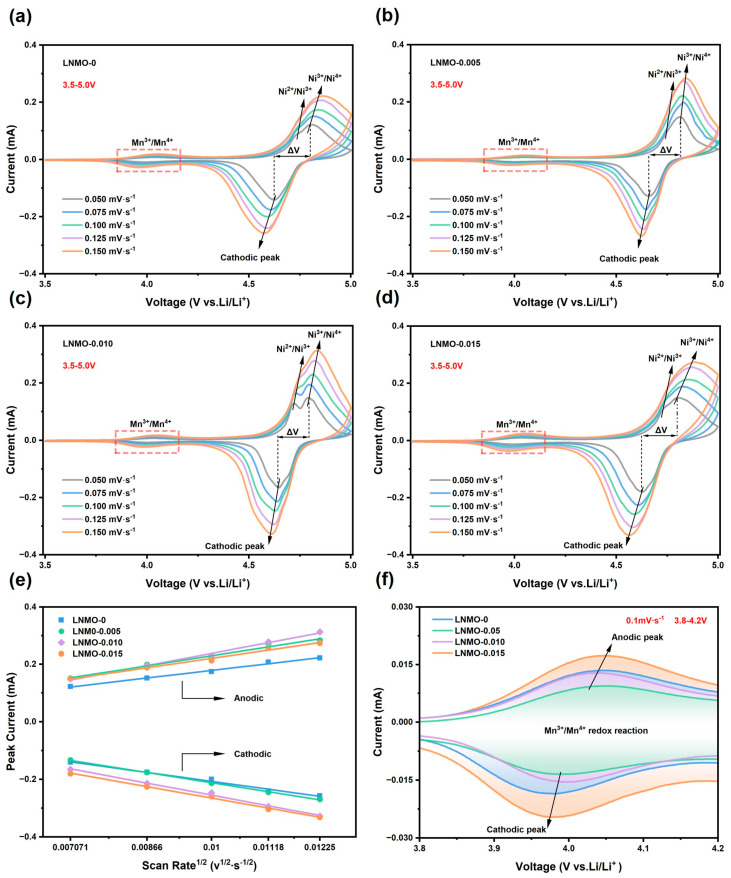
The electrochemical characterization of pristine and Mg^2+^/Cr^3+^ co-doped LNMO. (**a**) CV curves of LNMO-0 at different scan rates; (**b**) CV curves of LNMO-0.005 at different scan rates; (**c**) CV curves of LNMO-0.010 at different scan rates; (**d**) CV curves of LNMO-0.015 at different scan rates; (**e**) the linear relationship between the anodic and cathodic peak currents and the square root of the scan rate; (**f**) magnified CV curves of the four samples at the 4.0V peak under a scan rate of 0.100 mV/s.

To investigate the Li^+^ migration kinetics in pristine and Mg^2+^/Cr^3+^ co-doped LNMO, cyclic voltammetry (CV) measurements were performed on LNMO/Li half-cells over a voltage range of 3.5–5.0 V at scan rates of 0.05, 0.075, 0.10, 0.125, and 0.150 mV/s. As shown in Figure 5a–d, at a scan rate of 0.1 mV/s, all samples exhibit cathodic and anodic peaks near 4.6 V and 4.8 V, respectively. The redox peaks are split into two distinct peaks, corresponding to the Ni^2+^/Ni^3+^ and Ni^3+^/Ni^4+^ redox processes [63], with LNMO-0.010 displaying the most pronounced peak splitting. G.B. Zhong et al. reported that the potential difference between Ni^2+^/Ni^3+^ and Ni^3+^/Ni^4+^ redox peaks is greater in the Fd3m disordered phase than in the P4_3_32 ordered phase [64]. These findings suggest that the relative content of the Fd3m disordered phase among the samples follows the order LNMO-0.015 > LNMO-0.010 > LNMO-0.005 > LNMO-0, consistent with FTIR and Raman spectroscopy results. With the increase in scan rate, the anodic peak (de-lithiation) potentials of the four samples shift to higher values, while the cathodic peak (lithiation) potentials shift to lower values in Figure 5a–d. The potential difference between the anodic and cathodic peaks serves as a reliable indicator of the electrode material’s polarization degree. Table 2 compares the potential differences between the anodic and cathodic peaks of the four samples at different scan rates. At scan rates of 0.05, 0.125, and 0.150 mV/s, LNMO-0.010 exhibits the smallest potential difference, indicating the lowest electrode polarization among all samples. Figure 5e illustrates the linear relationship between the peak current (*i*_p_) and the square root of the scan rate (ν^1/2^). The anodic peak current increases with ν^1/2^, while the cathodic peak current decreases correspondingly, demonstrating a well-defined linear trend. This relationship is commonly employed to calculate the Li^+^ diffusion coefficient (D_Li_) using the Randles–Sevcik equation [65]:

*i*_p_ = 2.69 × 10^5^An^3/2^D_Li_Cν^1/2^ (1)

where A represents the surface area of the electrode (1.1304 cm^2^), n is the number of electrons transferred per reaction, and C is the volumetric concentration of LNMO (0.02378 mol∙cm⁻^3^). Table 3 presents the slopes of *i*_p_ versus ν^1/2^ and the corresponding D_Li_ values for Li^+^ extraction/insertion in the four samples. Mg^2+^/Cr^3+^ co-doping increases the Li^+^ extraction rate by an order of magnitude and also enhances the Li^+^ insertion rate, indicating that co-doping promotes faster Li^+^ diffusion. Among the co-doped samples, LNMO-0.010 exhibits the highest D_Li_ value, suggesting that a Mg^2+^/Cr^3+^ co-doping content of 0.010 improves the electronic conductivity and structural stability of LNMO, leading to superior electrochemical performance. A minor redox peak near 4.0 V, attributed to the Mn^3+^/Mn^4+^ redox process, is observed in all samples. Studies have shown that a reduced Mn^3+^ content mitigates the Jahn–Teller effect and decreases Mn dissolution in the electrolyte [54]. Figure 5f provides a magnified view of the Mn^3+^/Mn^4+^ redox peaks at 0.1 mV/s. At doping contents of 0.005 and 0.010, the peak areas at 4.0 V decrease, indicating a reduction in Mn^3+^ content. However, at a doping content of 0.015, the Mn^3+^ content surpasses that of the pristine sample, consistent with the Mn^3+^ relative content obtained from the XPS peak area ratios. This increase can be attributed to the charge compensation mechanism, wherein Mg^2+^/Cr^3+^ substitution for Mn^4+^ leads to an increase in Mn^3+^ content.

**Figure 6:**
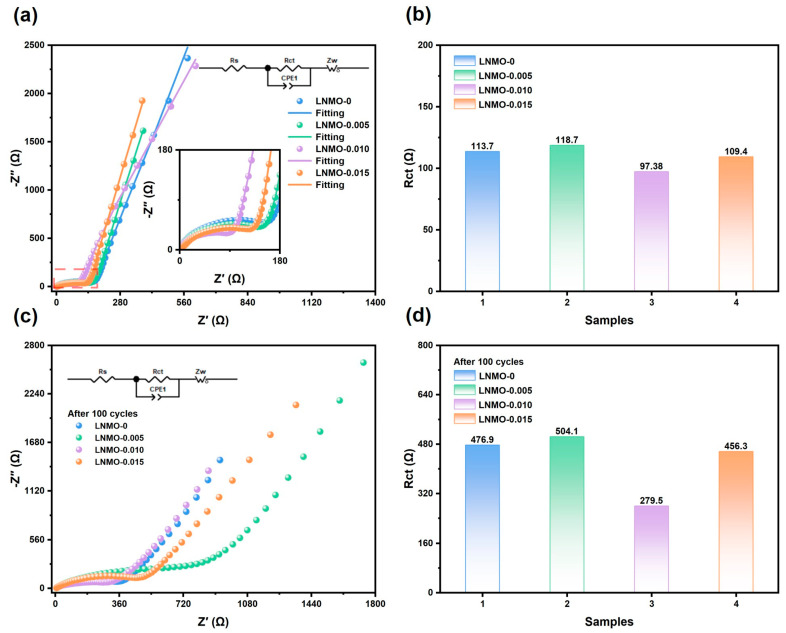
EIS curves (Nyquist plots) and R_ct_ values of pristine and Mg^2+^/Cr^3+^ co-doped LNMO. (**a**) Nyquist plots before cycling; (**b**) R_ct_ values before cycling; (**c**) Nyquist plots after 100 cycles at 0.5C; (**d**) R_ct_ values after 100 cycles at 0.5C.

To elucidate the beneficial effects of Mg^2+^/Cr^3+^ co-doping on LNMO, electrochemical impedance spectroscopy (EIS) was utilized to assess the impedance and interfacial behavior during the cycling process, activated by running five cycles before the test. Figure 6a,c displays the Nyquist plots obtained before cycling and after 100 cycles at 0.5C. All samples exhibit similar profiles, comprising a semicircle in the high-frequency region and an inclined line in the low-frequency region. The intercept in the high-frequency region corresponds to the ohmic resistance (R_s_), which is attributed to the contributions from the electrolyte, electrode material, and separator. The semicircle represents the charge transfer resistance (R_ct_) at the electrode/electrolyte interface, while the inclined line reflects the Warburg diffusion resistance (Z_w_), indicative of lithium-ion solid-state diffusion within the electrode material [66]. The Nyquist plots were fitted using software, achieving a high degree of correlation between the experimental data and fitted results. The equivalent circuit model employed for fitting and the derived R_ct_ values are shown in Figure 6a–d. Before cycling, the pristine LNMO (LNMO-0) exhibited a high impedance of 113.7 Ω, while the R_ct_ values of LNMO-0.005 (118.7 Ω) and LNMO-0.015 (109.4 Ω) were comparable to that of LNMO-0. In contrast, LNMO-0.010 demonstrated a significantly lower impedance of 97.38 Ω, representing a reduction of 14.35% relative to the pristine sample. The reduced impedance of LNMO-0.010 can be attributed to the introduction of Mg^2+^/Cr^3+^, which enhances conductivity and promotes a disordered structure. After 100 cycles at 0.5C, all samples exhibited increased R_ct_ values. The impedances of LNMO-0, LNMO-0.005, and LNMO-0.015 were approximately 500 Ω. However, LNMO-0.010 exhibited a markedly lower impedance of 279.5 Ω, which is only 58.61% of that of the pristine sample, indicating a substantial reduction. The inability of LNMO-0.005 and LNMO-0.015 to achieve similar impedance reductions is likely due to different factors. The low doping level in LNMO-0.005 failed to effectively substitute Ni and Mn sites, while the higher doping content in LNMO-0.015 led to an increased Mn^3+^ content, exacerbating Mn dissolution and interfacial side reactions. These findings highlight that Mg^2+^/Cr^3+^ co-doping at an optimal content of 0.010 effectively stabilizes the LNMO structure and the cathode/electrolyte interface, thereby enhancing the cycling performance.

**Figure 7:**
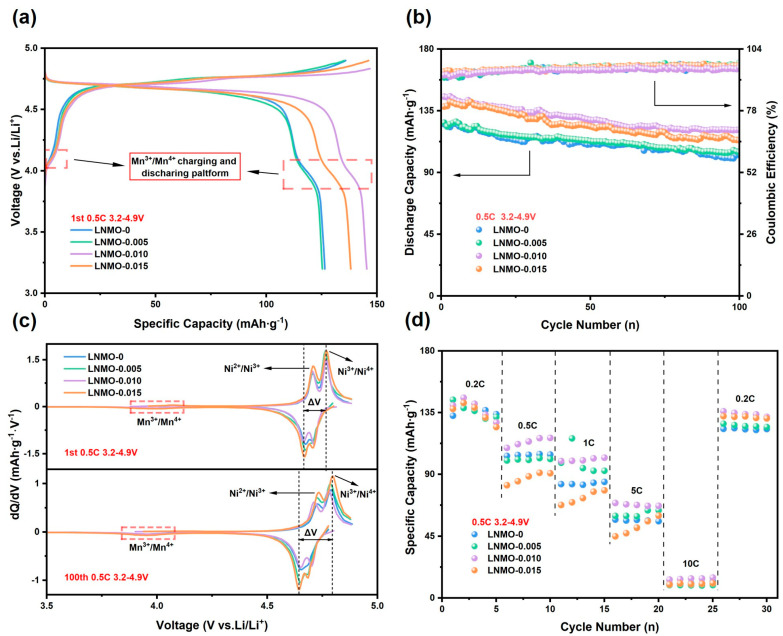
Electrochemical performance of pristine and Mg^2+^/Cr^3+^ co-doped LNMO. (**a**) Initial charge/discharge curves at 0.5C. (**b**) Cycling performance and Coulombic efficiency at 0.5C. (**c**) dQ/dV curves for the first and 100th cycles at 0.5C. (**d**) Rate performance.

The charge/discharge profile serves as a critical metric for assessing the electrochemical performance of LNMO materials. Figure 7a presents the initial charge/discharge curves of the four samples. All samples exhibit two distinct voltage plateaus at approximately 4.7 V, corresponding to the Ni^2+^/Ni^3+^ and Ni^3+^/Ni^4+^ redox couples. Additionally, a short voltage plateau appears at ~4.0 V in all four samples, attributed to the Mn^3+^/Mn^4+^ redox couple, indicating that the Fd3m disordered structure is predominant in all samples. This result is consistent with the findings from XRD, FTIR, Raman, and CV analyses. The Mn^3+^ content was calculated by dividing the discharge capacity within the 3.8~4.25 V range by the total discharge capacity [67]. Based on Figure 7a, the Mn^3+^ contents for LNMO-0, LNMO-0.005, LNMO-0.010, and LNMO-0.015 were determined to be 9.18%, 8.81%, 7.21%, and 9.33%, respectively. These values are in good agreement with the results obtained from XPS and CV. The reduced Mn^3+^ content in LNMO-0.010 effectively suppresses the Jahn–Teller effect and minimizes Mn dissolution, thereby achieving enhanced storage capacity.

The cycling performance of all samples was assessed through galvanostatic charge/discharge testing at 0.5C, as shown in Figure 7b. Prior to testing, each cell underwent 10 activation cycles at 0.2C to stabilize the electrodes. As presented in Figure 7b, the initial discharge capacities of LNMO-0, LNMO-0.005, LNMO-0.010, and LNMO-0.015 were 126.4, 125.3, 145.3, and 138.2 mAh∙g^−1^, respectively. After 100 cycles, the corresponding discharge capacities were 103.3, 104.84, 121.1, and 113.4 mAh∙g^−1^, with capacity retention rates of 82.45%, 82.93%, 83.32%, and 82.09%, respectively. The observed capacity degradation during cycling is primarily attributed to the progressive increase in charge transfer resistance (R_ct_) caused by interfacial reactions at the electrode/electrolyte interface. As shown in Figure 7b, Mg^2+^/Cr^3+^ co-doping notably enhances both the initial discharge capacity and cycling stability of the samples. This improvement is largely attributed to the higher binding energy of Mg/O and Cr/O bonds, which reinforces the structural stability of LNMO. Additionally, the three electrons in the third orbital of Cr^3+^ effectively mitigate the Jahn–Teller effect. Compared to LNMO-0.010, the performance of LNMO-0.005 is hindered by its insufficient doping level, which fails to fully capitalize on the advantages of Mg^2+^/Cr^3+^ co-doping. Conversely, LNMO-0.015 exhibits reduced cycling stability due to excessive doping, which increases Mn^3+^ content through charge compensation during Mg^2+^/Cr^3+^ substitution of Mn^4+^, thereby exacerbating side reactions and capacity fade. LNMO-0.010 exhibits better cycling performance, primarily due to the reduced Mn^3+^ content induced by doping and the increase in {100} crystal facets, which consequently reduce the proportion of {111} crystal facets. Previous studies have demonstrated that the cycling stability of LNMO is strongly influenced by surface orientation exposed to the electrolyte [35], and the exposed {111} crystal facets increase Mn dissolution and induce unstable interfacial behavior at higher voltages. This insight explains the rapid capacity decay observed in LNMO-0, whose particles predominantly expose {111} planes. Therefore, Mg^2+^/Cr^3+^ co-doping at an optimal concentration of 0.010 provides the most significant enhancement in both initial capacity and cycling stability, establishing LNMO-0.010 as the best-performing material. Figure 7b further depicts the Coulombic efficiency trends during cycling. The initial Coulombic efficiency is relatively low, which can be attributed to inevitable electrolyte decomposition and side reactions at the electrode/electrolyte interface under high voltage conditions [68]. However, a stable interfacial layer forms during cycling, leading to the subsequent stabilization of Coulombic efficiency in later cycles.

The differential capacity curve (dQ/dV) is a valuable tool for investigating the capacity fading mechanisms in LIBs, as the characteristic peaks on the curve correspond to the redox peaks observed in CV curves [69]. Figure 7c illustrates the dQ/dV curves of the four samples during the first and 100th cycles at 0.5C, revealing three pairs of redox peaks. The short plateau at approximately 4.0 V is associated with the Mn^3+^/Mn^4+^ redox couple, while the two redox peaks near 4.7 V are attributed to the Ni^2+^/Ni^3+^ and Ni^3+^/Ni^4+^ redox processes, respectively [70]. As shown in Figure 7c, after 100 cycles, the anodic peaks of all four samples shift to higher potentials while the cathodic peaks shift to lower potentials, and the potential difference (ΔV) increases, indicating an increased degree of electrode polarization after cycling. When considered alongside the observed increase in impedance after 100 cycles, it is evident that side reactions at the electrode/electrolyte interface and electrolyte decomposition play significant roles in this process. The deposition of decomposition products on the electrode surface obstructs Li^+^ transport and intensifies polarization, thereby contributing to capacity degradation in the LNMO samples.

To assess the rate performance of the prepared samples, the cells were first cycled at 0.2C for several cycles to activate the electrodes, followed by charge/discharge testing at sequential rates of 0.2C, 0.5C, 1C, 5C, and 10C, and finally returned to 0.2C. The corresponding rate performance curves are shown in Figure 7d. After the rate performance tests, the capacities of all samples nearly returned to their initial values, demonstrating the structural stability of the materials during rapid lithium insertion/extraction processes. The introduction of Cr^3+^ dopants extends the Li/O bond length, facilitating the separation of Li^+^ from O^2−^ and enhancing lithium-ion migration within the lattice [71]. Furthermore, spinel-type materials with high surface energy provide advantageous conditions for Li^+^ diffusion during charge/discharge processes [35]. As a result, LNMO-0.010, characterized by a lower Mn^3+^ content, fewer impurity phases, a higher proportion of Fd3m disordered structure, and prominent {100} crystal planes, exhibited superior capacities across different rates. At low rates of 0.2C and 0.5C, all samples displayed excellent performance. However, at higher rates of 5C and 10C, the capacities of the materials declined significantly. The rate capability of the materials is primarily governed by their morphology, particle size, and the availability of three-dimensional Li^+^ diffusion pathways. As shown in Figure 1c,g,k,o, LNMO-0, LNMO-0.005, LNMO-0.010, and LNMO-0.015 are composed of spherical aggregates formed by densely packed micro-octahedral structures. This compact porous morphology slows Li^+^ migration, leading to reduced capacities at high charge/discharge rates.

## 4. Conclusions

Based on the principle of crystal structure consistency, we successfully synthesized pristine disordered-phase LNMO samples using Ni/Mn bimetallic oxides as precursors. Structural characterizations (XRD, FTIR, Raman) and electrochemical performance (initial discharge capacity of 126.4 mAh·g^−1^) indicate the significant application potential of this approach. Furthermore, Mg^2+^/Cr^3+^ co-doped Ni/Mn bimetallic oxides were employed to prepare co-doped LNMO samples. Compared with the pristine LNMO, Mg^2+^/Cr^3+^ co-doping enhanced the degree of Ni/Mn disorder in the LNMO cathode material, reduced the surface Mn^3+^ content, and decreased the charge transfer resistance, thereby improving both the rate capability and cycling stability. Additionally, SEM analysis further revealed that Mg^2+^/Cr^3+^ co-doping facilitated the formation of high-surface-energy {100} crystal facets in LNMO grains, which promote lithium-ion migration. The 0.010 Mg^2+^/Cr^3+^ co-doped sample (LNMO-0.010) exhibited a significantly improved initial discharge capacity of 145.3 mAh·g^−1^ at 0.5C. Additionally, due to reduced electrode polarization and suppressed Mn^3+^ dissolution, this sample exhibited less capacity degradation compared to the pristine LNMO, achieving capacity retention of 83.32% after 100 cycles, thus demonstrating superior rate capability and cycling performance. This study offers an innovative and effective strategy for the synthesis and modification of LNMO cathode materials.

## Figures and Tables

**Table 1:** Rietveld refinement results for the pristine and Mg^2+^/Cr^3+^ co-doped LNMO.

Samples	a (Å)	V (Å^3^)	I_311_/I_400_	R_wp_
LNMO-0	8.174959	546.33	0.97	8.10
LNMO-0.005	8.173774	546.09	0.95	9.68
LNMO-0.010	8.168736	545.09	0.87	10.73
LNMO-0.015	8.168580	545.05	0.86	13.75

**Table 2:** Potential differences (ΔV, V) between anodic peaks (φ_pa_, V) and cathodic peaks (φ_pc_, V).

ν (mV∙s^−1^)	LNMO-0			LNMO-0.005			LNMO-0.010			LNMO-0.015		
	φ_pa_	φ_pc_	ΔV	φ_pa_	φ_pc_	ΔV	φ_pa_	φ_pc_	ΔV	φ_pa_	φ_pc_	ΔV
0.05	4.806	4.622	0.184	4.816	4.654	0.162	4.791	4.645	0.146	4.806	4.627	0.179
0.075	4.816	4.608	0.208	4.790	4.683	0.107	4.798	4.640	0.158	4.828	4.607	0.221
0.1	4.839	4.586	0.253	4.825	4.642	0.183	4.819	4.628	0.191	4.853	4.584	0.269
0.125	4.852	4.595	0.257	4.833	4.630	0.203	4.820	4.621	0.199	4.867	4.58	0.287
0.150	4.868	4.577	0.291	4.838	4.621	0.217	4.834	4.609	0.225	4.886	4.569	0.317

**Table 3:** The diffusion coefficients of Li^+^ in all samples.

Samples	Slope_pa_	Slope_pc_	Li-Deintercalation D_Li_ (cm^2^∙s^−1^)	Li-Intercalation D_Li_ (cm^2^∙s^−1^)
LNMO-0	1.976 × 10^−2^	−2.308 × 10^−2^	7.468 × 10^−12^	1.019 × 10^−11^
LNMO-0.005	2.636 × 10^−2^	−2.641 × 10^−2^	1.329 × 10^−11^	1.333 × 10^−11^
LNMO-0.010	3.180 × 10^−2^	−3.122 × 10^−2^	1.934 × 10^−11^	1.864 × 10^−11^
LNMO-0.015	2.458 × 10^−2^	−2.956 × 10^−2^	1.156 × 10^−11^	1.671 × 10^−11^

## Data Availability

The data presented in this study are available on request from the corresponding author.

## Supplementary Materials

The following supporting information can be downloaded at: https://www.mdpi.com/article/10.3390/nano15060429/s1, Figure S1: EDS mapping of the pristine and co-doped precursors. (a–c) EDS mapping of the pristine precursor; (d–h) EDS mapping of the precursor for the 0.005 Mg-Cr co-doped sample; (i–m) EDS mapping of the precursor for the 0.015 Mg-Cr co-doped sample.; Figure S2: Isothermal adsorption-desorption curves and pore size distribution profiles for both pristine and doped NMO samples were obtained. Figure S3: Ni XPS spectra of LNMO-0.005, LNMO-0.010, LNMO-0.015. (a) Ni XPS spectra of LNMO-0.005; (b) Ni XPS spectra of LNMO-0.010; (c) Ni XPS spectra of LNMO-0.015. Table S1: The BET specific surface area, total pore volume, and average pore diameter of both pristine and co-doped NMO samples were evaluated.
