# Supplementary material for: Mg2+ and Cr3+ Co-Doped LiNi0.5Mn1.5O4 Derived from Ni/Mn Bimetal Oxide as High-Performance Cathode for Lithium-Ion Batteries
Source: Nanomaterials (Basel). 2025 Mar 11;15(6):429. doi: 10.3390/nano15060429 (PMC11945260; doi:10.3390/nano15060429)
Supplement: Supplementary file 1 [file nanomaterials-15-00429-s001.zip › nanomaterials-3507436-supplementary.pdf]

Supporting Information

Mg^{2+} and Cr^{3+} Co-Doped $LiNi_{0.5}Mn_{1.5}O_4$ Derived from Ni/Mn Bimetal Oxide as High-Performance Cathode for Lithium-Ion Batteries

Dehua Ma [1,2], Jiawei Wang [1,2,3], Haifeng Wang [1,2,3,*], Guibao Qian [1,2], Xingjie Zhou [1,2], Zhengqing Pei [1,2], Kexin Zheng [1,2], Qian Wang [1,2] and Ju Lu [1,2]

1 College of Materials and Metallurgy, Guizhou University, Guiyang 550025, China; mdehua919@gmail.com (D.M.); jwwang@gzu.edu.cn (J.W.); 2414902764@163.com (G.Q.); 1916331338@163.com (X.Z.); 3163429870@163.com (Z.P.); 122754931@163.com (K.Z.); 1169979509@163.com (Q.W.); 2642656692@163.com (J.L.)

2 Guizhou Key Laboratory of Metallurgical Engineering and Process Energy Conservation, Guiyang 550025, China

3 Engineering Technology and Research Center of Manganese Material for Battery, Tongren 554300, China

* Correspondence: hfwang@gzu.edu.cn

1. Material Characterization and electrochemical analysis

1.1 Material Characterization

The nitrogen adsorption isotherm of NMO was measured using a Micromeritics ASAP 2460 surface area analyzer. X-ray powder diffraction (XRD) patterns were taken with a Bruker D8 Advance using Cu K α radiation (λ=0.1540 nm), The scanning rate was maintained at $5°·min^{-1}$, covering a 2θ range of 5° to 90°. The morphology was monitored by scanning electron microscope (ZEISS Sigma 360). The degree of Ni/Mn disordering was characterized by Fourier transform infrared spectroscopy (FTIR, Thermo Fisher Scientific Nicolet iS20) and Raman spectrum (Renishaw inVia). X-ray photoelectron spectroscopy (XPS) data were recorded using a Thermo Scientific K-Alpha spectrometer with Al K radiation.

1.2 Electrochemical analysis

Initially, LNMO powder, conductive carbon, and polyvinylidene fluoride (PVDF) binder were uniformly mixed in a weight ratio of 8:1:1 using N-methyl pyrrolidone (NMP). The resulting slurry was coated onto aluminum foil and vacuum-dried at 120°C for 12 hours. Discs with a diameter of 12 mm were then cut using a manual slicer. Button-type half-cells were assembled with LNMO as the working electrode, metallic lithium as the counter electrode, and a polyethylene membrane as the separator. All assembly procedures were carried out in an argon-filled glove box. A 5V high-voltage electrolyte ($LiPF_6$+EC+EMC, Guangdong Zhuguang New Energy Technology Co., Ltd.) was employed, and constant current charge-discharge tests as well as rate capability tests were conducted using a NEWARE battery testing system, $1C=147mAh·g^{-1}$. Cyclic voltammetry (CV) of the battery was measured using an electrochemical workstation (Donghua, DH7006). The voltage range is set from 3.5 to 5 V. Electrochemical impedance spectroscopy (EIS) tests were performed over a frequency range of 10^{-1} Hz to 10^{5} Hz on the electrochemical workstation.

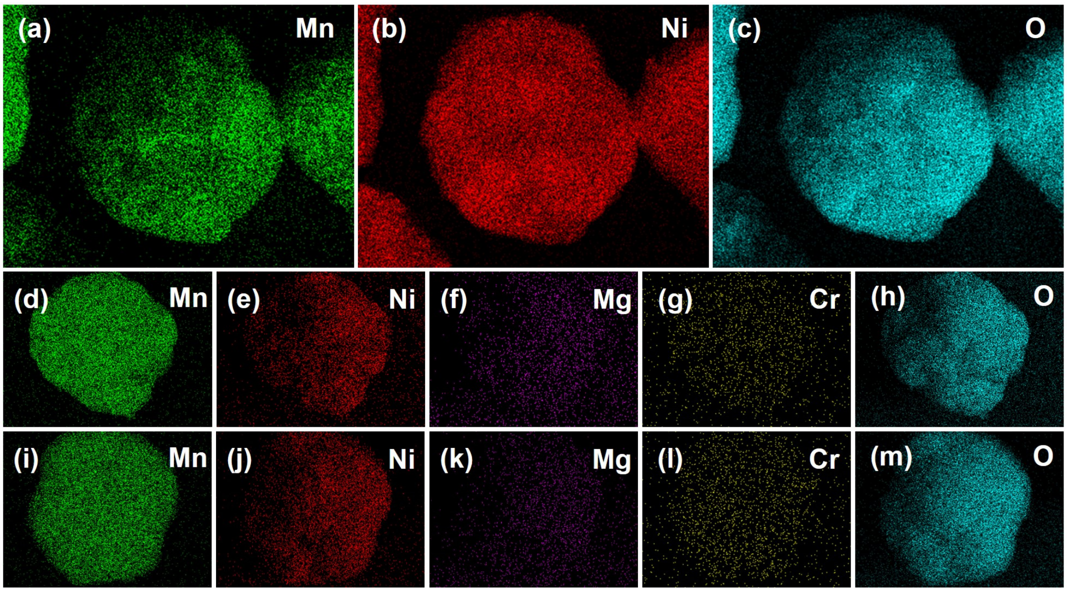

Figure S1. EDS mapping of the pristine and co-doped precursors. (a-c) EDS mapping of the pristine precursor; (d-h) EDS mapping of the precursor for the 0.005 Mg-Cr co-doped sample; (i-m) EDS mapping of the precursor for the 0.015 Mg-Cr co-doped sample.

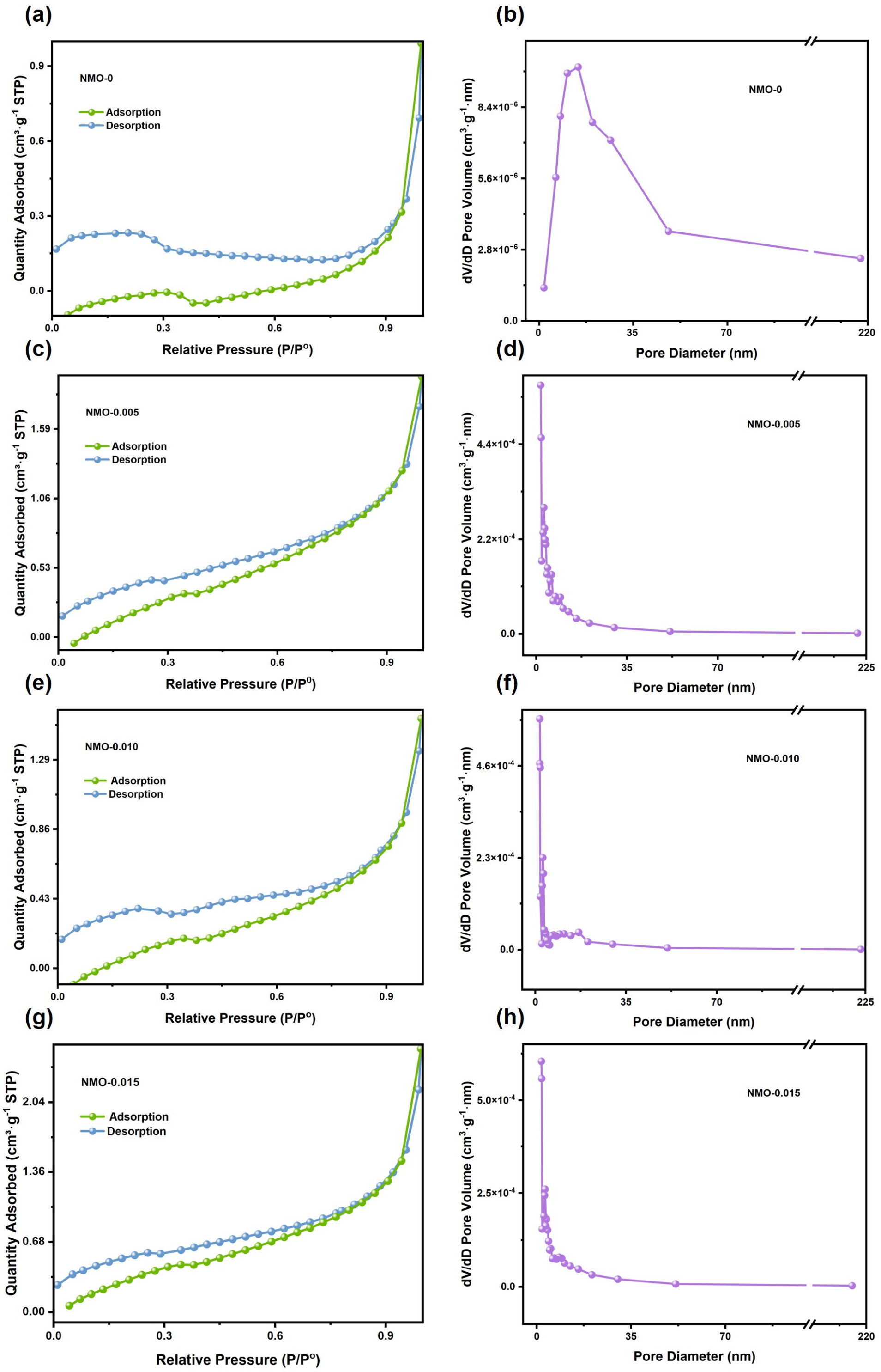

Figure S2. Isothermal adsorption-desorption curves and pore size distribution profiles for both pristine and doped NMO samples were obtained.

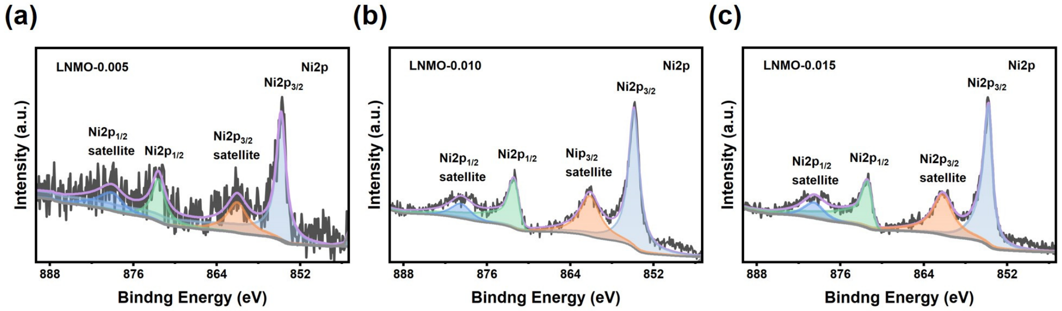

Figure S3. Ni XPS spectra of LNMO-0.005, LNMO-0.010, LNMO-0.015. (a) Ni XPS spectra of LNMO-0.005; (b) Ni XPS spectra of LNMO-0.010; (c) Ni XPS spectra of LNMO-0.015.

Table S1. The BET specific surface area, total pore volume, and average pore diameter of both pristine and co-doped NMO samples were evaluated.

Samples	BET Surface Area ($m^2 \cdot g^{-1}$)	Pore volume ($cm^3 \cdot g^{-1}$)	Average pore diameter (nm)
NMO-0	0.9162	0.001535	6.6999
NMO-0.005	1.5647	0.003080	7.8741
NMO-0.010	1.3454	0.002391	7.1089
NMO-0.015	1.9948	0.003959	7.9380
